# Mechanical power in pediatric acute respiratory distress syndrome: a PARDIE study

**DOI:** 10.1186/s13054-021-03853-6

**Published:** 2022-01-03

**Authors:** Anoopindar K. Bhalla, Margaret J. Klein, Vicent Modesto I Alapont, Guillaume Emeriaud, Martin C. J. Kneyber, Alberto Medina, Pablo Cruces, Franco Diaz, Muneyuki Takeuchi, Aline B. Maddux, Peter M. Mourani, Cristina Camilo, Benjamin R. White, Nadir Yehya, John Pappachan, Matteo Di Nardo, Steven Shein, Christopher Newth, Robinder Khemani, Pediatric Acute Lung Injury and Sepsis Investigators (PALISI) Network, Rossana Poterala, Analia Fernandez, Antonio Avila Vera, Nilda Agueda Vidal, Deheza Rosemary, Gonzalo Turon, Cecilia Monjes, Alejandro Siaba Serrate, Thomas Iolster, Silvio Torres, Pablo Castellani, Martin Giampieri, Claudia Pedraza, Luis Martin Landry, Maria Althabe, Yanina Vanesa Fortini, Simon Erickson, Samantha Barr, Sara Shea, Warwick Butt, Carmel Delzoppo, Alyssa Pintimalla, Alejandro Fabio Martinez Leon, Gustavo Alfredo Guzman Rivera, Philippe Jouvet, Guillaume Emeriaud, Mariana Dumitrascu, Mary Ellen French, Daniel Caro I, Carlos Acuna, Franco Diaz, Maria Jose Nunez, Yang Chen, Yurika Paola Lopez Alarcon, Ledys Maria Izquierdo, Byron Enrique Piñeres Olave, Pablo Vasquez Hoyos, Pierre Bourgoin, Florent Baudin, George Briassoulis, Stavroula Ilia, Matteo Di Nardo, Fabrizio Chiusolo, Nobuaki Shime, Shinichiro Ohshimo, Yoshiko Kida, Michihito Kyo, Swee Fong Tang, Chian Wern Tai, Lucy Chai See Lum, Ismail Elghuwael, Carlos Gil Escobar, Marta Sousa Moniz, Cristina Camilo, Tarek Hazwani, Nedaa Aldairi, Ahmed Al Amoudi, Ahmad Alahmadti, Yolanda Lopez Fernandez, Juan Ramon Valle, Lidia Martinez, Javier Pilar Orive, Vicent Modesto I Alapont, Marti Pons Odena, Alberto Medina, Susana Reyes Dominguez, Oguz Dursun, Ebru Atike Ongun, Fulya Kamit Can, Ayse Berna Anil, Jon Lillie, Shane Tibby, Paul Wellman, Holly Belfield, Joe Brierley, Troy E. Dominguez, Eugenia Abaleke, Yael Feinstein, James Weitz, Peter-Marc Fortune, Gayathri Subramanian, Claire Jennings, David Inwald, Calandra Feather, Rachel Agbeko, Angela Lawton-Woodhall, Karen McIntyre, Ryan Nofziger, Samir Latifi, Heather Anthony, Ron Sanders, Glenda Hefley, Manpreet Virk, Nancy Jaimon, Robinder Khemani, Christopher Newth, Anoopindar Bhalla, Jeni Kwok, Rica Morzov, Sidharth Mahapatra, Edward Truemper, Lucinda Kustka, Sholeen T. Nett, Marcy Singleton, J. Dean Jarvis, Nadir Yehya, Natalie Napolitano, Marie Murphy, Laurie Ronan, Ryan Morgan, Sherri Kubis, Elizabeth Broden, Rainer Gedeit, Kathy Murkowski, Katherine Woods, Mary Kasch, Yong Y. Han, Jeremy T. Affolter, Kelly S. Tieves, Amber Hughes-Schalk, Ranjit S. Chima, Kelli Krallman, Erin Stoneman, Laura Benken, Toni Yunger, James Schneider, Todd Sweberg, Aaron Kessel, Christopher L. Carroll, James Santanelli, Kate G. Ackerman, Melissa Cullimore, Courtney Rowan, Melissa Bales, W. Keith Dockery, Shirin Jafari-Namin, Dana Barry, Keary Jane’t, Shira Gertz, Bria Coates, Lawren Wellisch, Kiona Allen, Avani Shukla, Neal J. Thomas, Debbie Spear, Steven L. Shein, Margaret M. Parker, Daniel Sloniewsky, Christine Allen, Amy Harrell, Natalie Cvijanovich, Katri Typpo, Connor Kelley, Caroline King, Anil Sapru, Anna Ratiu, Neda Ashtari, Asumthia S. Jeyapalan, Alvaro Coronado-Munoz, Janet Hume, Dan Nerheim, Lincoln Smith, Silvia Hartmann, Erin Sullivan, Courtney Merritt, Awni Al-Subu, Andrea Blom, Deyin D. Hsing, Steve Pon, Jim Brian Estil, Richa Gautam, John S. Giuliano, Joana Tala

**Affiliations:** 1 Department of Anesthesiology and Critical Care Medicine, Children’s Hospital Los Angeles, Los Angeles, CA USA; 2 Department of Pediatrics, Keck School of Medicine, University of Southern California, Los Angeles, CA USA; 3 University and Polytechnic Hospital La Fe Valencia, Valencia, Spain; 4 Pediatric Intensive Care Unit, CHU Sainte-Justine, Department of Pediatrics, Université de Montréal, Montreal, Canada; 5 Division of Paediatric Critical Care Medicine, Department of Paediatrics, University Medical Center Groningen, Beatrix Children’s Hospital, University of Groningen, Groningen, The Netherlands; 6 Critical Care, Anaesthesiology, Peri-Operative & Emergency Medicine (CAPE), University of Groningen, Groningen, The Netherlands; 7 Hospital Universitario Central de Asturias, Oviedo, Spain; 8 Centro de Investigación de Medicina Veterinaria, Escuela de Medicina Veterinaria, Facultad de Ciencias de la Vida, Universidad Andres Bello, Santiago, Chile; 9 Departamento de Pediatría, Unidad de Paciente Crítico Pediátrico, Hospital El Carmen de Maipú, Santiago, Chile; 10 Instituto de Ciencias e Innovación ed Medicina (ICIM), Universidad del Desarrollo, Santiago, Chile; 11 Hospital Clínico La Florida, Santiago, Chile; 12 Department of Intensive Care Medicine, Osaka Women’s and Children’s Hospital, Osaka, Japan; 13 Pediatric Critical Care, University of Colorado School of Medicine, Aurora, CO USA; 14 Children’s Hospital Colorado, Aurora, CO USA; 15 Arkansas Children’s Hospital, University of Arkansas for Medical Sciences, Little Rock, AR USA; 16 PICU, Hospital de Santa Maria – CHULN, Lisbon, Portugal; 17 Penn State Health Children’s Hospital, Hershey, PA USA; 18 Department of Anesthesiology and Critical Care Medicine, Children’s Hospital of Philadelphia, Philadelphia, PA USA; 19 Paediatric Intensive Care Unit, University Hospital Southampton NHS Foundation Trust, Southampton, UK; 20 Faculty of Medicine, University of Southampton, Southampton, UK; 21 Pediatric Intensive Care Unit, Children’s Hospital Bambino Gesù, IRCCS, Rome, Italy; 22 Division of Pediatric Critical Care Medicine, Rainbow Babies and Children’s Hospital, Cleveland, OH USA

**Keywords:** Ventilators, Mechanical, Ventilator-induced lung injury, Critical care, Pediatrics

## Abstract

**Background:**

Mechanical power is a composite variable for energy transmitted to the respiratory system over time that may better capture risk for ventilator-induced lung injury than individual ventilator management components. We sought to evaluate if mechanical ventilation management with a high mechanical power is associated with fewer ventilator-free days (VFD) in children with pediatric acute respiratory distress syndrome (PARDS).

**Methods:**

Retrospective analysis of a prospective observational international cohort study.

**Results:**

There were 306 children from 55 pediatric intensive care units included. High mechanical power was associated with younger age, higher oxygenation index, a comorbid condition of bronchopulmonary dysplasia, higher tidal volume, higher delta pressure (peak inspiratory pressure—positive end-expiratory pressure), and higher respiratory rate. Higher mechanical power was associated with fewer 28-day VFD after controlling for confounding variables (per 0.1 J·min^−1^·Kg^−1^ Subdistribution Hazard Ratio (SHR) 0.93 (0.87, 0.98), *p* = 0.013). Higher mechanical power was not associated with higher intensive care unit mortality in multivariable analysis in the entire cohort (per 0.1 J·min^−1^·Kg^−1^ OR 1.12 [0.94, 1.32], *p* = 0.20). But was associated with higher mortality when excluding children who died due to neurologic reasons (per 0.1 J·min^−1^·Kg^−1^ OR 1.22 [1.01, 1.46], *p* = 0.036). In subgroup analyses by age, the association between higher mechanical power and fewer 28-day VFD remained only in children < 2-years-old (per 0.1 J·min^−1^·Kg^−1^ SHR 0.89 (0.82, 0.96), *p* = 0.005). Younger children were managed with lower tidal volume, higher delta pressure, higher respiratory rate, lower positive end-expiratory pressure, and higher PCO_2_ than older children. No individual ventilator management component mediated the effect of mechanical power on 28-day VFD.

**Conclusions:**

Higher mechanical power is associated with fewer 28-day VFDs in children with PARDS. This association is strongest in children < 2-years-old in whom there are notable differences in mechanical ventilation management. While further validation is needed, these data highlight that ventilator management is associated with outcome in children with PARDS, and there may be subgroups of children with higher potential benefit from strategies to improve lung-protective ventilation.

**Take Home Message**: Higher mechanical power is associated with fewer 28-day ventilator-free days in children with pediatric acute respiratory distress syndrome. This association is strongest in children <2-years-old in whom there are notable differences in mechanical ventilation management.

**Supplementary Information:**

The online version contains supplementary material available at 10.1186/s13054-021-03853-6.

## Background

Mechanical power is an estimate of the mechanical energy per minute that is applied to the respiratory system. Power is work divided by time where work is force exerted over a distance. The dissipation of excessive force or energy to the lung has been proposed to lead to ventilator-induced lung injury. Rather than separately assessing risk for lung strain or lung stress, mechanical power integrates these concepts, with respiratory rate and flow which may also contribute to ventilator induced lung injury [1–3]. In adults with acute respiratory distress syndrome (ARDS), higher mechanical power has been associated with higher mortality [4–7].

The original derivation of mechanical power introduced by Gattinoni et al., relied on transpulmonary pressure measurement and was applied only to patients on volume-controlled ventilation (constant flow) [8]. As transpulmonary pressure measurements and volume-controlled ventilation are not commonly used, it was initially difficult to test this concept in children. There are now several proposed surrogates or simplified equations for mechanical power allowing calculation on pressure-controlled ventilation with more routinely available data [9]. Nevertheless, because there are age-based differences in respiratory rate and tidal volume, methods need to consider normalization of these variables to adequately interpret mechanical power estimates in children.

Using simplified equations normalized to predicted body weight, we tested if higher mechanical power is associated with fewer ventilator-free days (VFD) in children with pediatric ARDS (PARDS). We also determined if the association between mechanical power and VFD differed by age, or hypoxemia severity. Finally, we sought to understand which ventilator management components included in mechanical power were most associated with VFDs in children and if mechanical energy (which does not include the age-dependent variable of respiratory rate) is associated with VFDs.

## Methods

We performed secondary analyses of the Pediatric Acute Respiratory Distress Syndrome Incidence and Epidemiology (PARDIE) study data [10]. PARDIE was an international prospective point prevalence study of children with newly diagnosed PARDS during 10 distinct study weeks in 2016 and 2017. Some PARDIE sites agreed a priori to contribute ventilator management data every 6 h during PARDS days 0–3 (PARDIE study V.2.) [11]. PARDIE study details are published separately [10–14]. The Children’s Hospital Los Angeles (CHLA) Institutional Review Board (IRB) (CHLA 16-0043) originally approved the PARDIE protocol. Except for one site, waiver of informed consent was granted by local IRBs.

### Study inclusion criteria

Children with PARDS managed on pressure-controlled, volume-controlled, pressure-regulated volume-controlled conventional ventilation within 24 h of PARDS diagnosis. *Study Exclusion Criteria:* Missing data, death, or transition to non-conventional ventilation limiting ability to calculate mechanical power at ≥ 2 of the 6-h measurement time points within 24 h of PARDS diagnosis. Extracorporeal membrane oxygenation support within 24 h of PARDS diagnosis.

Our primary objective was to evaluate the association between mechanical power and 28-day VFD IMV (days alive and free from invasive mechanical ventilation in the 28 days after PARDS diagnosis). Secondary objectives included assessing the association between 28-day VFD IMV and (1) mechanical power stratified by age and PARDS severity; (2) mechanical energy; (3) individual components of mechanical power (tidal volume [V_T_], respiratory rate [RR], peak inspiratory pressure [PIP], positive end-expiratory pressure [PEEP], delta pressure [PIP-PEEP]) [15]. We hypothesized high mechanical power was associated with fewer 28-day VFD IMV. We assessed ICU mortality, 28-day VFD (IMV and NIV) (accounting for invasive and non-invasive mechanical ventilation), and time to extubation in survivors as secondary outcomes.

Mechanical power was calculated at each 6-h measurement time point using the simplified equation proposed by Gattinoni et al., for volume-controlled ventilation and the surrogate equation proposed by Becher et al. for pressure-controlled ventilation (and pressure-regulated volume-controlled ventilation) [8, 16]. Other proposed methods of mechanical power were not considered as we did not have the required data variables, or reasonable surrogates [9]. PIP was substituted for plateau pressure in the volume-controlled equation (plateau pressure was rarely reported for children in this study), and mechanical power was normalized to predicted body weight [17, 18].

$${\text{Mechanical}}\;{\text{Power}}\;\left( {{\text{Volume - Controlled}}\;{\text{Ventilation}}} \right) \, ({\text{J}} \cdot {\text{min}}^{{ - {1}}} \cdot {\text{Kg}}^{{ - {1}}} \;{\text{predicted}}\;{\text{body}}\;{\text{weight}}) = \left( {{\text{V}}_{{\text{T}}} \left( {{\text{ml}}} \right) \times \left[ {{\text{PIP}}{-}{\text{delta}}\;{\text{pressure}}/{2}} \right] \times {\text{RR}} \times 0.0000{98}} \right)/{\text{predicted}}\;{\text{body}}\;{\text{weight}}\;\left( {{\text{kg}}} \right)$$

$${\text{Mechanical}}\;{\text{Power}}\left( {{\text{Pressure - Controlled}}\;{\text{Ventilation}}} \right)\;({\text{J}} \cdot {\text{min}}^{{ - {1}}} \cdot {\text{Kg}}^{{ - {1}}} \;{\text{predicted}}\;{\text{body}}\;{\text{weight}}) = \left( {{\text{V}}_{{\text{T}}} \left( {{\text{ml}}} \right) \times \left[ {{\text{PEEP}} + {\text{delta}}\;{\text{pressure}}} \right] \times {\text{RR}} \times 0.0000{98}} \right)/{\text{predicted}}\;{\text{body}}\;{\text{weight}}\;\left( {{\text{kg}}} \right)$$

Mechanical Energy was calculated using the previous equations, removing the RR component, at each 6-h measurement time point. We evaluated mechanical energy as mechanical power is dependent on RR, and there are age-dependent differences in physiologic RR.

Any child with ICU mortality was assigned zero 28-day VFD. A PARDS severity of illness score from a published predictive model for PARDS mortality and length of ventilation which adjusts for PaO_2_/FiO_2_ ratio, fluid balance, vasopressor-inotrope score, organ dysfunction, and immunocompromised conditions was used to control for initial disease severity in the analysis [12]. This predictive model was developed in the PARDIE cohort and validated in a separate cohort. Additional demographic, comorbidity, and management variables available from the PARDIE studies were considered for confounding or effect modification in the analysis (Online Additional file 1: Methods Supplement Table).

### Statistical analysis

Median parameters from ventilator data calculated using 6-h measurement times points over the first 24 h of PARDS were used for the analysis. Primary analyses: A multivariable competing risk regression model for risk of extubation at any given time (controlling for the competing risk of death, censored at 28 days of ventilation) and adjusting for center level effects using cluster robust standard errors was constructed to evaluate the association between mechanical power and 28-day VFD IMV [19]. Due to previously described limitations in multivariable modeling for the outcome of 28-day VFD directly, risk of extubation at any given time with the competing risk of death was primarily modeled, as a surrogate for 28-day VFD to ease interpretation of the results [20]. Based on biological plausibility, the multivariable model controlled for the pre-specified PARDS severity of illness score. Additional variables (site-specific, demographic, comorbidities, etc.) were considered as possible confounders for the multivariable model (Online Additional file 1: Methods Supplement Table). Variables were retained as confounders if they changed the mechanical power effect estimate by > 15%. Mechanical power is calculated based on mode of ventilation; therefore, ventilator mode was included in the model. Interaction terms were considered; however, there were no terms with a *p* ≤ 0.1. Similar multivariable models, logistic regression (ICU mortality), competing risk regression (28-day VFD [invasive and non-invasive ventilation]), and cox regression (time to extubation, survivors), were constructed for the secondary outcomes. All models were assessed for goodness of fit, multicollinearity, influence of outliers, and, as indicated, for confirmation of the proportional-hazards assumption as described in the Online Additional file 1: Methods Supplement.

We performed multiple sensitivity analyses to further explore and confirm our results. These are described briefly as follows with additional information available in the Online Additional file 1: Methods Supplement. Sensitivity Analysis 1: We performed a subgroup analysis excluding children with neurologic death from multivariable modeling for the outcome of ICU mortality as ventilator induced lung injury is less likely to be a significant contributor to death in these children. Sensitivity Analysis 2: Due to differences in mechanical power calculation based on mode of ventilation, we performed a subgroup analysis in children receiving only pressure-controlled ventilation. Sensitivity Analysis 3: We developed a propensity score for use of high mechanical power to inverse probability weight a competing risk regression model to confirm our main results. Sensitivity Analysis 4: We performed stratified analysis by age and PARDS severity to determine if these factors modified the relationship between mechanical power and outcome. Due to differences found by age, we described ventilator management by age and performed two additional sensitivity analyses. Sensitivity Analysis 5: We performed a subgroup analysis excluding children at highest risk of increased lower airway resistance (prematurity < 32 weeks estimated gestational age or bronchopulmonary dysplasia). Sensitivity Analysis 6: We developed a propensity score for use of high mechanical power specific to children < 2-years-old for inverse probability weighting of a competing risk regression model in children < 2-years-old. Sensitivity Analysis 7: To further explore the influence of respiratory rate on the association between mechanical power and outcome, we assessed the association between mechanical energy, which does not require respiratory rate, and outcome including a stratified analysis by age. Sensitivity Analyses 8: As mechanical power is comprised of multiple components of ventilator management; we examined the association between each component and outcome through univariable and multivariable modeling and a mediation analysis. We did not use delta pressure and PIP in any model together due to multicollinearity (tested using variance inflation factors and tolerance).

Additional details on data management, variable definitions, and the statistical analysis are available in the Online Additional file 1: Methods Supplement.

## Results

**Fig. 1:**
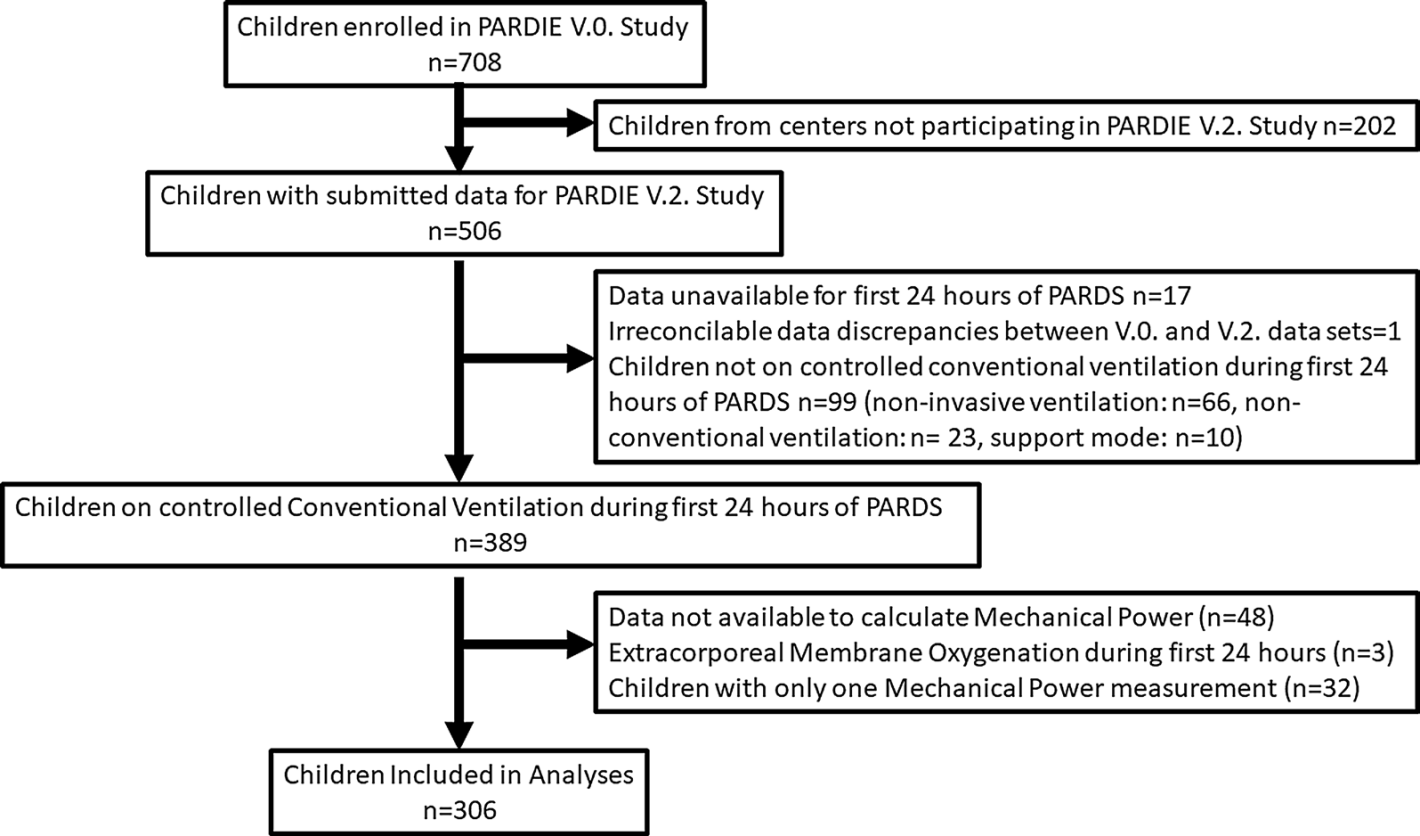
Enrollment Flow Chart. Non-conventional ventilation included high frequency oscillatory ventilation and airway pressure release ventilation. Data required to calculate mechanical power included predicted body weight (requires height to calculate) and ventilator management variables

There were 506 children enrolled in the PARDIE V.2. study, 306 of these children from 55 international PICU’s were included in this analysis (Fig. 1). ICU mortality was 16%, median 28-day VFD IMV were 19.1 (IQR 8.1, 23.4). Mode of ventilation was pressure-controlled in 39.5%, volume-controlled in 17.3%, pressure-regulated volume control in 30.4%, and multiple modes were used in 12.7%. The total respiratory rate was similar to the set ventilator rate for most children (median difference 0 [IQR 0, 4]). When examining children by quartiles of mechanical power, younger children, children with a higher oxygenation index, and children with a comorbidity of bronchopulmonary dysplasia were more often ventilated with higher mechanical power (Table 1). V_T_, RR, PIP, PEEP, and delta pressure increased across mechanical power quartiles as did the percentage of children on pressure-controlled ventilation. Median V_T_ was 8.8 ml/kg predicted body weight in the highest mechanical power quartile. Children with a higher mechanical power had a higher PCO_2_ and lower pH.

**Table 1:** Characteristics and outcomes of enrolled children by mechanical power quartiles

	Mechanical power quartiles (J·min^−1^·Kg^−1^ predicted body weight)			
	< 0.29	0.29–0.41	0.41–0.62	≥ 0.62
*n*	76	77	76	77
**Patient characteristics**				
Age (years)^a^	6.0 (1.4, 14.4)	2.3 (0.4, 8.2)	1.6 (0.4, 9)	0.9 (0.3, 4)
Male	47 (61.8%)	50 (64.9%)	52 (68.4%)	43 (55.8%)
Central/South America	7 (8.3%)	10 (11.9%)	8 (9.4%)	14 (17.3%)
Europe	14 (16.7%)	14 (16.7%)	5 (5.9%)	6 (7.4%)
Asia/Australia	4 (4.8%)	6 (7.1%)	4 (4.7%)	5 (6.2%)
North America	59 (70.2%)	54 (64.3%)	68 (80%)	56 (69.1%)
Comorbid condition	49 (58.3%)	48 (57.1%)	44 (51.8%)	52 (64.2%)
Bronchopulmonary Dysplasia	1 (1.2%)	1 (1.2%)	4 (4.7%)	13 (16.1%)
**PARDS characteristics**				
Direct Lung Injury	60 (70.6%)	54 (63.5%)	58 (66.7%)	64 (76.2%)
Oxygenation Index^b^	4.9 (3.7, 7.7)	7.6 (5.0, 12.1)	8.6 (5.3, 13.7)	11.1 (6.8, 16.1)
NIV at Diagnosis	5 (6.6%)	8 (10.4%)	7 (9.2%)	9 (11.7%)
**Ventilator management parameters**				
Mechanical ventilation mode pressure controlled	18 (23.7%)	29 (37.7%)	29 (38.2%)	45 (58.4%)
PRVC	22 (29%)	20 (26%)	30 (39.5%)	21 (27.3%)
Volume controlled	24 (31.6%)	21 (27.3%)	8 (10.5%)	0 (0%)
Multiple modes	12 (15.8%)	7 (9.1%)	9 (11.8%)	11 (14.3%)
V_T_ (ml/kg predicted body weight)	6.7 (5.6, 7.6)	7.2 (5.8, 8.6)	7.7 (7, 8.9)	8.8 (7.4, 10)
PIP (cm H_2_O)	22 (19, 24)	26 (23, 29)	28 (25, 32)	32 (30, 36)
Delta pressure (cm H_2_O)	14 (12, 18)	18 (14, 20)	20 (17, 22)	23 (20, 27)
RR (bpm)	19 (15, 24)	22 (18, 28)	24 (20, 30)	30 (25, 35)
PEEP (cm H_2_O)	7 (6, 8)	8 (6, 10)	8 (7, 10)	8 (7, 11)
pCO_2_^c^	42 (37, 50)	45 (38, 54)	48 (40, 55)	54 (43, 61)
pH^c^	7.36 (7.30, 7.42)	7.35 (7.30, 7.40)	7.31 (7.25, 7.39)	7.33 (7.25, 7.38)
**Outcomes**				
28-day VFD (IMV)	21.4 (13.6, 24.7)	21.7 (15.0, 24.2)	18.1 (4.7, 21.9)	15.6 (0, 21.6)
28-day VFD (IMV and NIV)	21.2 (8.7, 24.4)	20.8 (12.4, 23.5)	17.4 (0.3, 21.9)	14.8 (0, 21.0)
Length of ventilation (survivors)	5.3 (3.0, 12.5)	5.9 (3.7, 10.4)	8.2 (4.2, 14.1)	9.3 (5.2, 13.9)
ICU mortality	7 (9.2%)	8 (10.4%)	12 (15.8%)	22 (28.6%)

Data are presented as count and percentage or median and interquartile range. The median values per subject for the first 24 h of pediatric acute respiratory distress syndrome (PARDS) are reported. IMV: invasive mechanical ventilation. NIV: non-invasive ventilation. PEEP: positive end-expiratory pressure. PIP: peak inspiratory pressure. PRVC: pressure-regulated volume-controlled ventilation. RR: respiratory rate. VFD: ventilator-free days. V_T_: tidal volume

^a^Two children with missing age (*n* = 1 quartile 3 (Q3), *n* = 1 Q4)

^b^Three children with missing median oxygenation index (*n* = 2 Q3, *n* = 1 Q4)

^c^PCO_2_ and pH values were available for *n* = 66 Q1 (1 child with pH but not PCO_2_), *n* = 69 Q2, *n* = 74 Q3, *n* = 74 Q4

^d^Two children with missing data on length of ventilation (*n* = 1 Q3, *n* = 1 Q4)

### Primary outcome

Higher mechanical power was associated with fewer 28-day VFD IMV in univariable analysis after adjusting for center level effects (per 0.1 J·min^−1^·Kg^−1^ predicted body weight SHR 0.89 [0.84, 0.94], *p* < 0.0001) and in multivariable modeling after controlling for center-level effects and confounding variables including the pre-specified PARDS severity of illness score, a comorbidity of bronchopulmonary dysplasia, height, PCO_2_, and mode of ventilation (per 0.1 J·min^−1^·Kg^−1^ predicted body weight SHR 0.93 [0.87, 0.98], *p* = 0.013) (Table 2).

**Table 2:** The association between mechanical power and 28-day ventilator-free days and ICU mortality

	28-day Ventilator-free days (IMV)			ICU mortality		
	*n*	SHR (95% CI)	*p* value	*n*	OR (95% CI)	*p* value
**Univariable models**						
*Entire cohort*						
Mechanical power	304	0.89 (0.84, 0.94)	< 0.001	306	1.26 (1.12, 1.41)	< 0.001
**Multivariable models**						
*Entire cohort*						
Mechanical Power	304	0.93 (0.87, 0.98)	0.013	306	1.12 (0.94, 1.32)	0.20
*Subgroup excluding children with neurologic death*						
Mechanical Power	291	0.91 (0.86, 0.97)	0.002	293	1.22 (1.01, 1.46)	0.036
*Age Subgroups*						
< 2 years: Mechanical Power	149	0.89 (0.82, 0.96)	0.005	151	1.19 (0.97, 1.46)	0.087
≥ 2 years: Mechanical Power	153	0.97 (0.84, 1.12)	0.68	153	0.94 (0.69, 1.29)	0.71
*PARDS severity subgroups*						
Resolved/Mild PARDS: Mechanical Power	163	0.90 (0.80, 1.03)	0.12	164	1.00 (0.73, 1.37)	0.99
Moderate/severe PARDS: Mechanical Power	141	0.94 (0.87, 1.03)	0.18	142	1.25 (0.99, 1.59)	0.059

All estimates are per 0.1 J·min^−1^·Kg^−1^ predicted body weight change in mechanical power. All models adjusted for center-level effects. Multivariable models control for the pre-specified pediatric acute respiratory distress syndrome (PARDS) severity of illness score, a comorbidity of bronchopulmonary dysplasia, height, PCO_2_, and mode of ventilation. The pre-specified PARDS severity of illness score adjusts for immunocompromised conditions, the 6-h PaO_2_/FiO_2_ ratio, and the fluid balance, vasopressor-inotrope score, and organ dysfunction on the first day of PARDS. There were 2 children without length of ventilation data that were excluded from the VFD models. There were 2 children missing age excluded from the age subgroup models. IMV: invasive mechanical ventilation

### Secondary outcomes

Mechanical power was associated with higher ICU mortality in univariable analysis after adjusting for center level effects (per 0.1 J·min^−1^·Kg^−1^ predicted body weight OR 1.26 [1.12, 1.41], *p* < 0.0001) but was no longer statistically significant in multivariable analysis after controlling for center level effects and confounding variables (per 0.1 J·min^−1^·Kg^−1^ predicted body weight OR 1.12 [0.94, 1.32], *p* = 0.20) (Table 2). Higher mechanical power was associated with fewer 28-day VFD (IMV and NIV) in univariable (per 0.1 J·min^−1^·Kg^−1^ predicted body weight SHR 0.89 [0.85, 0.94], *p* < 0.001) and multivariable analysis after controlling for confounding variables (per 0.1 J·min^−1^·Kg^−1^ predicted body weight SHR 0.92 [0.87, 0.98], *p* = 0.006) (Additional file 1: Table S1). In survivors, higher mechanical power was associated with a longer time to extubation in univariable (per 0.1 J·min^−1^·Kg^−1^ predicted body weight HR 0.96 [0.91, 1.00], *p* = 0.067) and multivariable analysis (per 0.1 J·min^−1^·Kg^−1^ predicted body weight HR 0.96 [0.90, 1.02], *p* = 0.19) although the results were not statistically significant (Additional File 1: Table S1).

### Sensitivity analysis 1

**Fig. 2:**
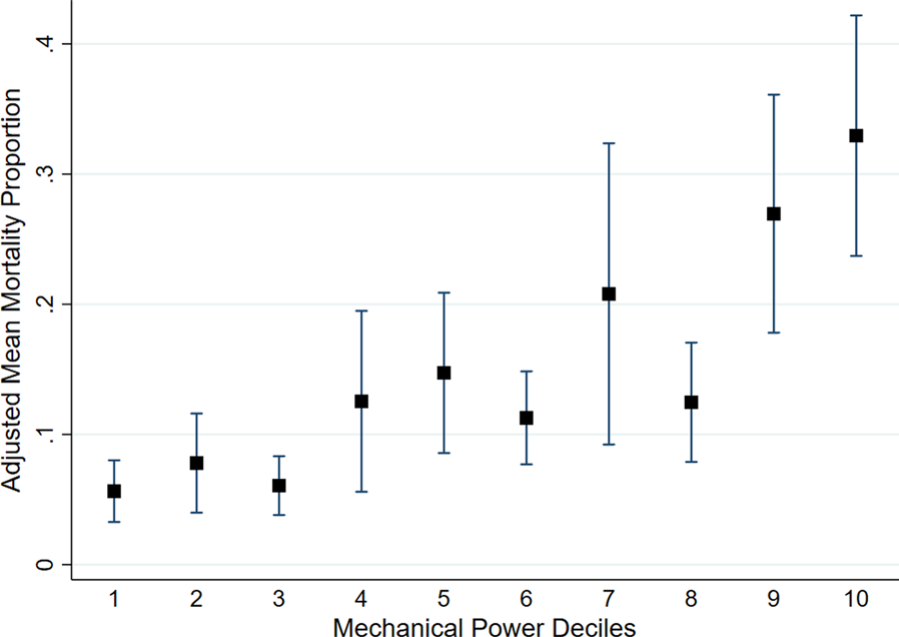
Adjusted Mean Mortality Proportion by Mechanical Power Deciles (excluding children who died from neurologic injury). Black squares represent mean, and whiskers represent 95% confidence interval. The mean mortality proportion was calculated after multivariable adjustment for the pre-specified pediatric acute respiratory distress syndrome (PARDS) severity of illness score, a comorbidity of bronchopulmonary dysplasia, height, PCO_2_, and mode of ventilation. The pre-specified PARDS severity of illness score adjusts for immunocompromised conditions, the 6-h PaO_2_/FiO_2_ ratio, and the fluid balance, vasopressor-inotrope score, and organ dysfunction on the first day of PARDS

In a sensitivity analysis, excluding the 14 children who died due to neurologic reasons, higher mechanical power was independently associated with higher ICU mortality in multivariable analysis (per 0.1 J·min^−1^·Kg^−1^ predicted body weight OR 1.22 [1.01, 1.46], p = 0.036) (Table 2, Fig. 2).

### Sensitivity analysis 2

In multivariable analysis limited to children on either pressure-controlled or pressure-regulated volume-controlled ventilation, the association between higher mechanical power and fewer 28-day VFD IMV remained (per 0.1 J·min^−1^·Kg^−1^ predicted body weight SHR 0.90 [0.83, 0.97], *p* = 0.005).

### Sensitivity analysis 3

A propensity score developed for the use of high mechanical power included the following variables: the pre-specified PARDS severity of illness score, median 24-h oxygenation index, height, use of corticosteroids, pressure-controlled mode of ventilation, and a comorbidity of bronchopulmonary dysplasia (Additional file 1: Table S2, Additional file 1: Figure S1). Higher mechanical power was associated with fewer 28-day VFD (per 0.1 J·min^−1^·Kg^−1^ predicted body weight SHR 0.91 [0.84, 0.99], *p* = 0.025) and higher ICU mortality (per 0.1 J·min^−1^·Kg^−1^ predicted body weight OR 1.24 [1.09, 1.43], *p* = 0.002) after inverse probability weighting for the developed propensity score and controlling for center level effects.

### Sensitivity analysis 4

Stratifying by the median age of 2-years-old, for children < 2-years-old ICU mortality was 14.6%, compared to 17.6% ICU mortality for those ≥ 2-years-old. In subgroup analysis, the association between higher mechanical power and fewer 28-day VFD remained only in children < 2-years-old (per 0.1 J·min^−1^·Kg^−1^ predicted body weight SHR 0.89 [0.82, 0.96], *p* = 0.005) (Table 2). We did not find an association between mechanical power and 28-day VFD or ICU mortality in children > 2-years-old or within PARDS severity subgroups (mild/resolved and moderate/severe) (Table 2).

Given the age-based effects on the association between mechanical power and outcome, we explored age-based differences in patient characteristics and ventilator management. There were regional and comorbidity differences by age, and younger children more frequently had direct lung injury (Table 3). Younger children were managed with lower V_T_, higher delta pressure, higher RR, lower PEEP, and higher PCO_2_. Outcomes were similar across age quartiles.

**Table 3:** Characteristics of children by age quartiles

	Age quartiles (years)^a^			
	< 0.5	0.5 – 2	2 – 8.3	≥ 8.3
*n*	75	76	77	76
**Patient characteristics**				
Central/South America	13 (17.3%)	5 (6.6%)	11 (14.3%)	5 (6.6%)
Europe	15 (20%)	7 (9.2%)	9 (11.7%)	4 (5.3%)
Asia/Australia	6 (8%)	4 (5.3%)	6 (7.8%)	0 (0%)
North America	41 (54.5%)	60 (79%)	51 (66.2%)	67 (88.2%)
Any comorbid condition	38 (50.7%)	39 (51.3%)	49 (63.6%)	49 (64.5%)
Bronchopulmonary Dysplasia	4 (5.3%)	12 (15.8%)	1 (1.3%)	0 (0%)
Prematurity (< 32 weeks EGA)	10 (13.3%)	14 (18.4%)	4 (5.2%)	1 (1.3%)
Oncologic Disease	0 (0%)	2 (2.6%)	7 (9.1%)	12 (15.8%)
Chronic Respiratory Disease	6 (8.0%)	24 (31.6%)	21 (27.3%)	22 (29.0%)
Congenital Cardiac Disease	9 (12.0%)	13 (17.1%)	6 (7.8%)	3 (4.0%)
Acquired Cardiac Disease	7 (9.3%)	12 (15.8%)	4 (5.2%)	6 (7.9%)
Neuromuscular Disease	4 (5.3%)	8 (10.5%)	20 (26.0%)	20 (26.3%)
**PARDS characteristics**				
Direct Lung Injury	58 (77.3%)	62 (81.6%)	54 (70.1%)	40 (52.6%)
Oxygenation Index^b^	7.0 (5.0, 11.3)	7.4 (4.8, 13.8)	8.1 (4.8, 13)	7.3 (4.5, 13.7)
NIV at Diagnosis	1 (1.3%)	5 (6.6%)	9 (11.7%)	14 (18.4%)
**Ventilator management parameters**				
Mechanical ventilation mode pressure controlled	31 (41.3%)	24 (31.6%)	37 (48.1%)	28 (36.8%)
PRVC	21 (28%)	27 (35.5%)	18 (23.4%)	26 (34.2%)
Volume controlled	11 (14.7%)	12 (17.1%)	14 (18.2%)	15 (19.7%)
Multiple modes	12 (16%)	12 (15.8%)	8 (10.4%)	7 (9.2%)
V_T_ (ml/kg predicted body weight)	6.5 (5.6, 8.5)	7.5 (6.3, 8.6)	7.7 (7.0, 8.9)	7.9 (6.6, 9.7)
PIP (cm H_2_O)	27 (23, 31)	28 (24, 31)	25 (22, 30)	28 (23, 32)
Delta pressure (cm H_2_O)	20 (17, 24)	20 (15, 23)	18 (14, 20)	18 (14, 23)
RR (bpm)	30 (25, 35)	26 (24, 30)	20 (18, 25)	18 (16, 21)
PEEP (cm H_2_O)	7 (6, 8)	8 (7, 9)	8 (7, 11)	9 (7, 12)
Mechanical power (J·min^−1^∙Kg^−1^ predicted body weight)	0.47 (0.33, 0.66)	0.48 (0.35, 0.69)	0.36 (0.26, 0.52)	0.35 (0.26, 0.48)
Mechanical energy (J·Kg^−1^ predicted body weight)	0.016 (0.011, 0.022)	0.019 (0.014, 0.026)	0.018 (0.013, 0.022)	0.019 (0.015, 0.026)
PCO_2_ (mm Hg)^c^	56 (48, 66)	50 (41, 56)	43 (38, 52)	40 (35, 46)
pH^c^	7.31 (7.25, 7.39)	7.34 (7.26, 7.38)	7.35 (7.29, 7.42)	7.36 (7.31, 7.42)
**Outcomes**				
Length of ventilation (survivors)	6.1 (4.5, 9.8)	7.4 (4.1, 14.2)	6.5 (4.0, 14.8)	9 (3.7, 13)
28-day VFD (IMV)	21.0 (14.6, 23.4)	18.5 (7.5, 23.5)	19.4 (2.0, 23.3)	18.2 (4.0, 23.8)
28-day VFD (IMV and NIV)	20.6 (14.0, 22.9)	18.0 (5.3, 23.2)	18.2 (0, 23.2)	16.6 (0, 22.4)
ICU Mortality	9 (12%)	13 (17.1%)	14 (18.2%)	13 (17.1%)

Data are presented as count and percentage or median and interquartile range. The median values for the first 24 h of pediatric acute respiratory distress syndrome (PARDS) were reported. EGA: estimated gestational age. IMV: invasive mechanical ventilation. NIV: non-invasive mechanical ventilation. PEEP: positive end-expiratory pressure. PIP: peak inspiratory pressure. PRVC: pressure-regulated volume-controlled ventilation. RR: respiratory rate. VFD: ventilator-free days. V_T_: tidal volume

^a^2 children missing age

^b^3 children missing median oxygenation index (*n* = 1 quartile 1 (Q1), *n* = 1 Q2, *n* = 1 Q3)

^c^PCO_2_ and pH measurements available for *n* = 67 Q1, *n* = 64 Q2 (1 child with pH but not PCO_2_), *n* = 71 Q3, *n*  = 73 Q4

^d^2 children in Q2 missing length of ventilation

### Sensitivity analyses 5 and 6

Due to differences noted by age in patient and PARDS characteristics, we performed two additional sensitivity analyses in children < 2-years-old. We first excluded children with prematurity < 32 weeks estimated gestational age or bronchopulmonary dysplasia as these children are known to have increased lower airways resistance which may contribute to use of higher mechanical power. We found that higher mechanical power remained associated with fewer 28-day VFD (per 0.1 J·min^−1^·Kg^−1^ predicted body weight SHR 0.88 [0.81, 0.97], *p* = 0.009) after controlling for confounding variables when these children were excluded from the analysis (Additional file 1: Table S3). Second, we developed a propensity score for use of high mechanical power in children < 2-years-old. Higher mechanical power remained associated with fewer 28-day VFD (per 0.1 J·min^−1^·Kg^−1^ predicted body weight SHR 0.92 [0.85, 0.999], *p* = 0.047) in children < 2-years-old after inverse probability weighting for this propensity score and controlling for center level effects (Additional file 1: Table S3).

### Sensitivity analysis 7

The two highest quartiles of mechanical energy were associated with fewer 28-day VFD compared to the lowest quartile (quartile 3 [Q3]: SHR 0.62 [0.48, 0.79], *p* < 0.0001 and Q4: SHR 0.65 [0.47, 0.92], *p* = 0.014 versus Q1) but not higher ICU mortality in multivariable models controlling for center-level effects and other confounding variables (Additional file 1: Table S4). In an age-stratified analysis, unlike mechanical power, mechanical energy was not higher in children < 2-years-old (Table 3). Nevertheless, the association between mechanical energy and fewer 28-day VFD was only present in children < 2-years-old.

### Sensitivity analysis 8

In univariable analyses, individual components of mechanical power (V_T_, PEEP, PIP, delta pressure) were all associated with fewer 28-day VFD except for RR (Additional file 1: Table S5). However, in multivariable modeling, only a PIP ≥ 31 cm H_2_O referenced to a PIP < 22 cm H_2_O (SHR 0.67 [0.46, 0.97], *p* = 0.033) was independently associated with fewer 28-day VFD after controlling for center-level effects, RR, V_T_, PEEP, and other confounding variables (Additional file 1: Tables S6, S7). In analysis using structural equation modeling, no individual component of mechanical power (V_T_, PEEP, PIP, RR, or delta pressure) mediated the effect of mechanical power on either 28-day VFD or ICU mortality (Additional file 1: Table S8).

## Discussion

In a diverse international cohort of children, we found that use of high mechanical power (normalized to predicted body weight) while on conventional mechanical ventilation during early PARDS was associated with fewer 28-day VFD. While ICU mortality and 28-day VFD did not differ significantly by age, the association between higher mechanical power and fewer 28-day VFD was strongest in children < 2-years-old. Additionally, when excluding children who died primarily due to neurologic causes, we found an association between higher mechanical power and ICU mortality. No specific ventilator management component was deemed a statistically significant mediator in the association between mechanical power and 28-day VFD or ICU mortality, which suggests the potential importance of the combination variable of mechanical power to capture risk.

While we hypothesized that the relationship between mechanical power and 28-day VFD may be age-dependent, we did not expect that the association would be strongest in children < 2-years-old. We found that compared to older children, children < 2-years-old were managed with lower PEEP, lower V_T_ per predicted body weight, higher RR, and higher delta pressure. Some of these findings are predictable, as physiologic RR is higher in younger children, and we have previously found in this cohort that younger children are more likely to be managed with a PEEP lower than recommended by the ARDS Network Lower PEEP/Higher FiO_2_ grid [11]. We hypothesize this may be due to clinician concern with high PEEP in young children due to higher chest wall compliance. Furthermore, younger children may also have higher resistance in their lower airways, which will be even higher if they have concurrent bronchopulmonary dysplasia (a confounding variable in modeling). This will result in higher delta pressure (PIP—PEEP) with each breath related to resistive work. However, if ventilator induced lung injury is primarily driven by elastic components of the respiratory system (PEEP, V_T_, driving pressure [plateau pressure—PEEP]), then we would have expected that higher mechanical power related to higher airway resistance would not be injurious. Rather, even after normalizing V_T_ to predicted body weight and adjusting for height in multivariable modeling, we found the opposite in that children < 2-years-old had the strongest relationship between higher mechanical power and worse outcome. This relationship remained consistent in sensitivity analyses removing children with prematurity or bronchopulmonary dysplasia and adjusting for a propensity score developed for use of high mechanical power in children < 2-years-old.

An alternate explanation is that when lower PEEP is used in younger children, there is more atelectrauma and inadequate lung recruitment. This then results in lower lung compliance, with higher delta pressure to achieve the same V_T_. Hence, the elevated delta pressures in younger children may reflect higher lung stress. It is noteworthy that not only are younger children ventilated with a higher delta pressure, but they also have lower V_T_ per predicted body weight and higher PCO_2_ which may support this theory. There are age-based differences in minute ventilation and higher levels of physiologic dead space, even in intubated children, which may also contribute to the use of higher mechanical power in younger children [21, 22]. Knowing the static condition with plateau pressure in these children could have helped differentiate the contributions of the elastic and resistive components, but our data highlight that clinicians either do not measure or record plateau pressure in children very often.

The respiratory rate component of mechanical power may result in different thresholds of harm based on age [23]. For this reason, we evaluated mechanical energy, and unlike mechanical power, we found that mechanical energy was not higher in younger children. Nevertheless, the relationship between higher mechanical energy and fewer 28-day VFD was significant only in children < 2-years-old. This implies that the respiratory rate component of mechanical power is not a primary factor capturing risk. While there are some pre-clinical data which suggests that younger animals are less susceptible to the risks of volutrauma than older animals, there may be biologic plausibility that younger children are at higher risk of harm if they are managed with high mechanical power, particularly if this higher power is coming from elevated delta pressure [24–27]. Transpulmonary pressures may be higher for a given delta pressure in a young child, compared to an older child, due to the young child’s higher chest wall compliance. This results in more energy transmission to the lung rather than the chest wall. Therefore, routine management practices in younger children, lower PEEP because of higher chest wall compliance and higher delta pressures due to increased airway resistance, may be harmful and should be reconsidered and further studied.

Although none of the individual ventilator management components mediated the effect of mechanical power on either 28-day VFD IMV or ICU mortality, PIP was most strongly associated with 28-day VFD IMV in multivariable modeling. This is in contrast to a recent adult ARDS meta-analysis which found that driving pressure contributed to much of the effect of mechanical power on outcome, with a smaller component related to respiratory rate [4]. While possible that driving pressure may have performed better than delta pressure in our cohort, for risk of injury, PIP appears to give similar information to delta pressure as children are rarely managed with very high PEEP levels [11, 28]. On the other hand, higher RR was not associated with worse outcome in children, as mechanical energy was associated with 28-day VFD IMV. This is similar to another study in a general cohort of children requiring mechanical ventilation which found that higher mechanical energy was associated with longer duration of ventilation, although this study did not find an association with mechanical power [23]. Our results suggest that mechanical power may be a useful metric to identify risk of ventilator induced lung injury when V_T_ is normalized to predicted body weight, without further adjustment for physiologic differences in respiratory rate.

Our study is observational in nature, and therefore, we cannot conclude that lowering mechanical power will result in better outcomes for children with ARDS. Nevertheless, the concept of mechanical power may be an important construct as we try to prioritize the importance of individual components of ventilatory support in children with ARDS. It may be that strategies to decrease mechanical power should focus on identifying if mechanical power decreases with lung recruitment and titrating PEEP. If the lung recruits with the application of PEEP, compliance should improve, and a lower delta pressure can be used to achieve the same tidal volume. If the lung does not recruit, then reducing mechanical power will require lower minute ventilation with more tolerance for permissive hypercapnia or alternative therapies to improve alveolar ventilation by decreasing physiologic dead space. This highlights the need for a more robust understanding of the interaction between ventilator management strategies and specific pathophysiologic states in children with ARDS. Future research should seek to further characterize the association between mechanical power and harm in children by also investigating if there is a threshold below which mechanical power is no longer harmful and if this threshold may differ by PARDS severity, concepts that are supported by research on mechanical power in adults with ARDS [6, 29].

There are several limitations to this study. Data submission was voluntary and despite numerous inquiries to participating sites, some data remained missing. However, a small percentage of children (13%) were excluded due to missing data. Although we attempted to control for confounding variables, it is possible the associations that we found between high mechanical power and worse outcomes are related to unmeasured confounding. There were notable differences in patient characteristics by age which we attempted to control for with a propensity score; however, most of these patient characteristics did not seem to be associated with use of high mechanical power. It is possible other unmeasured differences in the cohort by age may have confounded the association between mechanical power and outcome. For the minority of children on volume-controlled ventilation, we substituted PIP for plateau pressure in the Gattinoni et al. equation for mechanical power. Lack of reporting for plateau pressure prevented us from rigorously differentiating resistive from elastic components of work. We normalized mechanical power to predicted body weight, but there is no consensus about the correct approach to normalization [17]. Alternative methods for normalization of mechanical power have been proposed (such as respiratory system compliance), but we did not have the data to compute these equations [5]. Additional study may be helpful to determine normal values for mechanical power by age. Although all children were on controlled ventilation, we were unable to quantify mechanical power related to spontaneous effort as plateau pressure was not available, and we did not assess the contribution to mechanical power of breaths above the set ventilator rate [30]. This may have led to an underestimation of mechanical power in some children. However, we expect this would have biased our results towards the null. The Becher equation for mechanical power is known to overestimate mechanical power, and more children were on pressure-controlled ventilation in the higher mechanical power quartiles [31]. We have previously shown that pressure-controlled ventilation is more often used than other modes in children with more severe PARDS [11]. However, higher mechanical power related to use of the Becher equation would have likely biased our results to the null. In contrast, we found in analysis limited to children with mechanical power calculated with the Becher equation that the association between higher mechanical power and fewer 28-day VFD remained.

## Conclusions

Higher mechanical power is associated with worse outcomes in children with PARDS. This association is strongest in children < 2-years-old where there are notable differences in mechanical ventilation management. However, no specific component of ventilator management mediated this association. While further validation is needed, these data highlight that ventilator management is associated with outcome in children with PARDS, and there may be subgroups of children with higher potential benefit from strategies to improve lung-protective ventilation.

## Supplementary Information

**Additional file 1**.** Table S1**: Multivariable Analysis for Secondary Outcomes of 28-day VFD (IMV and NIV) and Time to Extubation in Survivors. ** Table S2**: Propensity Score Multivariable Model for Use of High Mechanical Power (≥ 0.62 J min^−1^ Kg^−1^ predicted body weight). ** Table S3**: Additional Sensitivity Analyses Limited to the Subgroup of Children <2 years of Age. ** Table S4**: Multivariable models for the Association between Mechanical Energy and 28-day Ventilator-Free Days and Mortality. ** Table S5**: The Univariable Association between each Ventilation Management Component of Mechanical Power and 28-day Ventilator-Free Days. ** Table S6**: Multivariable Model for 28-day Ventilator-Free Days considering all Ventilator Management Components of Mechanical Power (with Delta Pressure) (n = 304). ** Table S7**: Multivariable Model for 28-day Ventilator-Free Days considering all Ventilator Management Components of Mechanical Power (with Peak Inspiratory Pressure) (n=304). ** Table S8**: Structural Equation Modeling. ** Figure S1**: Distribution of Propensity Scores

## Data Availability

Data set will be publicly available in 2024.

## Abbreviations

ICU: Intensive Care Unit
IMV: Invasive Mechanical Ventilation
NIV: Non-invasive Mechanical Ventilation
PARDS: Pediatric Acute Respiratory Distress Syndrome
PEEP: Positive End-Expiratory Pressure
PIP: Peak Inspiratory Pressure
RR: Respiratory Rate
VFD: Ventilator Free Days
V_T_: Tidal Volume
