# Supplementary material for: Mechanical power in pediatric acute respiratory distress syndrome: a PARDIE study
Source: Crit Care. 2022 Jan 3;26:2. doi: 10.1186/s13054-021-03853-6 (PMC8722295; doi:10.1186/s13054-021-03853-6)
Supplement: Supplementary file 1 — Additional file 1. Table S1: Multivariable Analysis for Secondary Outcomes of 28-day VFD (IMV and NIV) and Time to Extubation in Survivors. Table S2: Propensity Score Multivariable Model for Use of High Mechanical Power (≥ 0.62 J min−1 Kg−1 predicted body weight). Table S3: Additional Sensitivity Analyses Limited to the Subgroup of Children <2 years of Age. Table S4: Multivariable models for the Association between Mechanical Energy and 28-day Ventilator-Free Days and Mortality. Table S5: The Univariable Association between each Ventilation Management Component of Mechanical Power and 28-day Ventilator-Free Days. Table S6: Multivariable Model for 28-day Ventilator-Free Days considering all Ventilator Management Components of Mechanical Power (with Delta Pressure) (n = 304). Table S7: Multivariable Model for 28-day Ventilator-Free Days considering all Ventilator Management Components of Mechanical Power (with Peak Inspiratory Pressure) (n=304). Table S8: Structural Equation Modeling. Figure S1: Distribution of Propensity Scores [file 13054_2021_3853_MOESM1_ESM.docx]

**Title:** Mechanical power in pediatric acute respiratory distress syndrome: A PARDIE Study

**Journal:** Critical Care

**Authors:**

1. Anoopindar K. Bhalla MD, MSCI
2. Margaret J. Klein, MS
3. Vicent Modesto i Alapont, MD, PhD
4. Guillaume Emeriaud, MD
5. Martin C.J. Kneyber, MD, PhD
6. Alberto Medina, MD
7. Pablo Cruces, MD
8. Franco Diaz, MD
9. Muneyuki Takeuchi, MD, PhD
10. Aline B. Maddux, MD, MSCS
11. Peter M. Mourani, MD
12. Cristina Camilo, MD
13. Benjamin R. White, MD, MA
14. Nadir Yehya, MD, MSCE
15. John Pappachan, MD
16. Matteo Di Nardo, MD
17. Steven Shein, MD
18. Christopher Newth, MD
19. Robinder Khemani, MD, MSCI
20. Pediatric Acute Lung Injury and Sepsis Investigators (PALISI) Network

**Corresponding Author:**

Anoopindar Bhalla, MD, MSCI

Department of Anesthesiology and Critical Care Medicine, Children’s Hospital Los Angeles

Keck School of Medicine, University of Southern California

Email: [abhalla@chla.usc.edu](mailto:abhalla@chla.usc.edu)

**Additional File 1 (Methods Supplement, Supplemental Tables, Figures)**

**METHODS SUPPLEMENT**

We performed secondary analysis of the Pediatric Acute Respiratory Distress Syndrome Incidence and Epidemiology (PARDIE) study data.(1) PARDIE was an international prospective point prevalence study that enrolled 744 children from 145 pediatric intensive care units (PICUs) with newly diagnosed pediatric acute respiratory distress syndrome (PARDS) during 10 distinct study weeks in 2016 and 2017 (PARDIE V.0.). Some PARDIE sites agreed a priori to contribute ventilator management data, every 6 hours during PARDS days 0-3 (PARDIE study V.2.).(2)

Creation of the PARDIE V.2. Analytic Dataset

PICUs provided data approximately every 6 hours for respiratory support settings (non-invasive mechanical ventilation [NIV], conventional mechanical ventilation, and high frequency oscillatory ventilation [HFOV]), respiratory monitoring, blood gases, and neuromuscular blockade administration during the first 3 days of PARDS. Children on controlled conventional ventilation (pressure-controlled, volume-controlled, pressure regulated volume control) in the first 24 hours of PARDS were included for this study analysis. Children were excluded if they did not have the data to calculate mechanical power on ≥2 data points on controlled conventional ventilation within the first 24 hours of PARDS. Conventional ventilation measurements from times points with an endotracheal tube leak >20% (defined as (Inspiratory tidal volume (V_T_) – Expiratory V_T_)/Inspiratory V_T_) were considered unreliable and excluded from the analysis. Children may have not had the data to calculate mechanical power at ≥2 measurement time points in the first 24 hours due to several reasons: missing data, endotracheal tube leak >20%, transition to non-conventional ventilation, or death. Children placed on extracorporeal membrane oxygenation (ECMO) within the first 24 hours of PARDS were also excluded.

For each measurement time point (roughly every 6 hours), PARDS severity was calculated using Oxygenation Index (OI=(Mean Airway Pressure [MAP] x FiO_2_ x 100)/PaO_2_) or when PaO_2_ was not available and SpO_2_ was ≤97%, Oxygen Saturation Index (OSI=[MAP x FiO_2_ x 100]/SpO_2_). OSI was converted to an OI equivalent as previously described.(3)

Data Imputation and Cleaning

Data imputation was performed with different methods which were chosen based on the physiology of the missing variable and availability of similar data variables.

When PARDS severity could not be calculated due to either missing data or an SpO_2_ >97%, the mean of the oxygenation metric in the previous measurement time point and the subsequent measurement time point were used to impute OI when available.

When data was not available to calculate an endotracheal tube leak at a measurement time point, the leak was assumed to be <20% unless the child had other measurement time points with a leak >20%. Most children (86%) were ventilated with a cuffed endotracheal tube supporting this approach.

Any blood gas (arterial, capillary, or venous) was accepted for pH and PCO_2_. There were 41 children without a blood gas within the first 24 hours of PARDS. For children without a blood gas, PCO_2_ was imputed. We have previously shown in this cohort that median PCO_2_ differs by delta pressure.(2) Therefore, we first categorized each child by their delta pressure (Peak Inspiratory Pressure (PIP) – PEEP) as either higher or lower than the median delta pressure value for the cohort. Then, within each strata of delta pressure, the median PCO_2_ for other children in that strata was used to impute PCO_2_ for children missing PCO_2_. PCO_2_ values presented in the descriptive analyses do not include imputed PCO_2_.

Queries were sent to site investigators throughout the study to resolve inconsistent data. However, when inconsistencies were found in the data, two investigators (A.B. and M.K.) corrected minor errors in the data using the V.0. PARDIE dataset for adjudication. For example, every 6 hours mean airway pressure, SpO_2_, and FiO_2_ data were recorded in both the V.0. and V.2. PARDIE datasets. If in the V.2. dataset an SpO_2_ of 19% was recorded and the saturation in the V.0. dataset for the same time period was 91%, we assumed a data entry error and corrected the value in the V.2. dataset to the V.0. SpO_2_. If correction was not easily agreed upon, or the data was severely incongruent, these measurement time points were excluded from the analysis.

Variable Definition

A previously published predictive score for mortality and length of ventilation in PARDS which adjusts for 6-hour PaO_2_/FiO_2_ ratio, fluid balance (first day of PARDS), vasopressor-inotrope score (first day of PARDS), organ dysfunction (first day of PARDS), and immunocompromised conditions was used in the analysis to control for initial disease severity.(4) PARDS severity was defined using established cut-points.(5)

28-day ventilator free days (VFD IMV) were calculated as the days alive and free of invasive mechanical ventilation in the first 28 days after PARDS diagnosis. All children with ICU mortality were given 28-day VFD of zero. Children were followed for 90 days after PARDS diagnosis however this was not considered in 28-day VFD. 28-day ventilator free days (VFD IMV and NIV) were calculated as the days alive and free of invasive and non-invasive mechanical ventilation in the first 28 days after PARDS diagnosis. Non-invasive ventilation was defined as either continuous positive airway pressure (CPAP) or Bilevel positive airway pressure (BiPAP).

Predicted body weight was estimated using the McLaren method and age-appropriate validated growth curves (<2 years of age: World Health Organization and ≥2 years of age: Centers for Disease Control).(6, 7) Calculation of predicted body weight required an available height measurement. Delta pressure was used in the analyses because Pplat, and thus driving pressure, was not recorded for most children. The set conventional ventilator rate was used as the respiratory rate in the analysis. Time to extubation was defined as >48 hours without invasive mechanical ventilation. PARDIE sites reported the cause of death for enrolled children and we identified children that died due to neurologic injury (brain death, cerebral edema, etc.).

Statistical Analysis

Primary Analysis: Median values for 6-hour time measurement points during the first 24 hours of PARDS were calculated for each ventilator management variable and for OI for each child and used for the analysis. We reported descriptive variables without significance testing based on STROBE (strengthening the reporting of observational studies in epidemiology) guidelines.

A multivariable competing risk regression model for risk of extubation at any given time (controlling for the competing risk of death, censored at 28 days of ventilation) and adjusting for center-level effects using cluster robust standard errors was constructed to evaluate the association between mechanical power and 28-day VFD IMV.(8, 9) Although risk of extubation at any given time given the competing risk of death was modeled, we used the surrogate 28-day VFD IMV for ease of interpretation of the results. This is due to previously described limitations in multivariable modeling with the outcome of 28-day VFD.(10) Multiple variables (demographic, site specific, comorbidities, etc.) were considered as possible confounders for the model (Methods Table). Variables were retained as confounders in the multivariable model if they changed the effect estimate for mechanical power by >15%. Based on biological plausibility, the multivariable model controlled for the pre-specified PARDS severity of illness score. Mechanical power is calculated based on mode of ventilation; therefore, ventilator mode was included as a potential confounder in the model. Mechanical power interaction terms were considered but there were no terms with a p value ≤0.1. Risk of multicollinearity was assessed through correlation between variables, examination of standard errors, and examination of variance inflation factors and tolerance. We identified outliers and evaluated their impact on model performance. To evaluate model fit over time for the mechanical power covariate we plotted the Schoenfeld residuals against the unique failure times (time to extubation). The plots did not show any evidence of a non-constant local average over time.(11) To test the proportional-hazards assumption we confirmed that the mechanical power coefficient was time invariant by testing the time interaction as well as testing mechanical power as a time-varying covariate. The interaction was non-significant and there was no observed issue of post-failure covariate values for patients who fail from the competing risk of death.

Secondary Outcomes: We developed a univariable mixed effects logistic regression model for the secondary outcome of ICU mortality adjusting for random center-level effects. We then developed a multivariable mixed effects logistic regression model for the secondary outcome of ICU mortality including adjustment for confounding variables identified from the multivariable model for the primary outcome of 28-day VFD IMV. Additional variables were considered (Methods Table) but none met the threshold of changing the effect estimate for mechanical power by >15%. Mechanical power interaction terms were considered but none had a p value <0.1. As model results were similar with and without clustering by site, goodness of fit was considered using a Hosmer-Lemeshow test without consideration of clustering by site. For the secondary outcome of 28-day VFD IMV and NIV we used a competing risk regression model (controlling for the competing risk of death, censored at 28 days of invasive and non-invasive ventilation) adjusting for center-level effects using cluster robust standard errors and including adjustment for confounding variables from the multivariable model for the primary outcome of VFDs. We again considered additional variables for multivariable modeling but not met the threshold for inclusion. Mechanical power interaction terms were considered but none had a p value <0.1. We assessed the proportional-hazard assumption and goodness of fit using time-varying coefficients and Schoenfeld residuals. For the secondary outcome of time to extubation in survivors, we used a cox regression model adjusting for center-level effects using cluster robust standard errors including adjustment for confounding variables from the multivariable model for the primary outcome of VFDs. Again, although considered, additional variables for multivariable modeling did not met the threshold for inclusion. Mechanical power interaction terms were considered but none had a p value <0.1. The proportional-hazards assumption was tested with Schoenfield residuals. Goodness of fit was assessed with Cox-Snell residuals.

Sensitivity Analysis 1: We performed a sensitivity analysis excluding children with neurologic death from the multivariable mortality model as the mortality risk is less likely to be related to ventilator-induced lung injury in these children.

Sensitivity Analysis 2: We performed a sensitivity analysis excluding children managed with volume-controlled ventilation due to differences in calculation of mechanical power based on mode of ventilation.

Sensitivity Analysis 3: We developed a propensity score to estimate the probability for use of high mechanical power (highest quartile of mechanical power). Variables with a p<0.05 for the association with high mechanical power were retained in the multivariable logistic regression propensity score model. Two-way interaction terms were assessed and included if p<0.1. Balance of covariates was assessed through graphics (Additional Data File Figure 1). The developed propensity score was used to inverse probability weight a competing risk regression model for 28-day VFD IMV and a logistic regression model for ICU mortality. These models adjusted for center-level effects using cluster robust standard errors. Potentially confounding variables that occurred after the decision to use high mechanical power in the first 24 hours (use of inhaled Nitric Oxide, high frequency oscillatory ventilation, neuromuscular blockade, prone positioning, ECMO after the first 24 hours of PARDS), were evaluated for inclusion in the multivariable model. None of these variables changed the association between mechanical power and outcome by >15% and were therefore not included. Model assumptions and goodness of fit were tested as previously described for other similar models.

Sensitivity Analysis 4: To determine if age or PARDS hypoxemia severity modified the association between mechanical power and 28-day VFD IMV, stratified analyses by each variable were performed. We used the median age in the cohort, 2 years, to stratify the age analysis. Due to differences noted by age subgroup, we performed a descriptive analysis to explore how mechanical ventilation practices differ by age quartiles.

Sensitivity Analysis 5: As increased lower airway resistance may be a reason for high mechanical power, we performed a sensitivity analysis limited to children <2 years old without a comorbid condition of either bronchopulmonary dysplasia or prematurity <32 weeks estimated gestational age.

Sensitivity Analysis 6: Given there were age-dependent differences in type of lung injury and comorbid conditions, a propensity score was developed for the use of high mechanical power in children <2 years old (with a similar methodology to the previously described propensity score developed for high mechanical power in the entire cohort). The propensity score for use of high mechanical power in the children <2 years old was then used to inverse probability weight a competing risk regression model for 28-day VFD IMV.

Sensitivity Analysis 7: As mechanical energy does not include the age-dependent variable of respiratory rate, we performed additional analyses assessing mechanical energy in multivariable modeling to determine the association between mechanical energy and 28-day VFD IMV and ICU mortality. The relationship between mechanical energy and outcome was not linear therefore we modeled mechanical energy as a categorical variable. Models were developed and tested with similar methodology to previous described models.

Sensitivity Analysis 8: To assess which components of mechanical ventilation (PIP, PEEP, RR, V_T_, delta pressure) primarily contribute to outcome, we assessed the association between each variable and 28-day VFD IMV in a series of univariable models. We then constructed a multivariable model including each component of mechanical power to assess the independent relationship of each variable with 28-day VFD IMV. We did not use delta pressure and peak inspiratory pressure in a model together due to multicollinearity (tested using variance inflation factors and tolerance). Variables were modeled as categorical as there was not a linear relationship between variables and outcome. Confounding variables from the primary analysis were included in multivariable modeling. Additional confounding variables and interaction terms were considered but none met the previously described thresholds for inclusion. Model assumptions and goodness of fit was assessed as previously described for similar models.

We performed mediation analyses using structural equation modeling to assess the indirect effect of each ventilator management component of mechanical power on 28-day VFD IMV. The lavaan package in R was used with the DWLS estimator with bia-corrected and accelerated confidence intervals using 100 bootstrapped samples.

All statistical analyses were performed with STATA 15 (StataCorp LLC, College Station) and RStudio Version 1.4.1106 (RStudio, Boston, MA). A p value of <0.05 was considered significant.

**Methods Supplement Table:** Data Variables available from the PARDIE Study Considered as Possible Confounders

| **Variables** |
| --- |
| Age |
| Gender |
| Weight |
| Height |
| Body Mass Index |
| Race |
| Ethnicity |
| Admission Season |
| PARDS Risk Factors (by type and direct/indirect) |
| Comorbid Conditions (by type) |
| Post-operative Status |
| 12-hour PRISM III Score |
| PICU Region |
| Number of PICU Beds |
| Middle Income versus High Income Country |
| Average PICU Admissions Per Year |
| ICU Physician In-House 24 Hours |
| PICU Fellowship |
| Respiratory therapist involved in ventilator management |
| Ventilator decisions made primarily by the attending physician |
| Chest Radiograph Bilateral Infiltrates |
| Cuffed Endotracheal Tube |
| PARDS Severity (resolved, mild, moderate, severe) |
| Oxygenation Index |
| pH |
| PCO_2_ |
| Prone positioning |
| Inhaled Nitric Oxide |
| High Frequency Oscillatory Ventilation |
| Extracorporeal Membrane Oxygenation |
| Non-invasive Ventilation prior to Intubation |
| Neuromuscular Blockade |
| Diuretics |
| Corticosteroids |
| Vasoactive Inotrope Score |
| Fluid Balance |
| Blood product Transfusion (for each component ml and yes/no) |
| PELOD-2 Score (Total score and by organ system) |

**METHODS SUPPLEMENT REFERENCES**

1. Khemani RG, Smith L, Lopez-Fernandez YM, Kwok J, Morzov R, Klein MJ, et al. Paediatric acute respiratory distress syndrome incidence and epidemiology (PARDIE): an international, observational study. Lancet Respir Med. 2019;7(2):115-28.

2. Bhalla AK, Klein MJ, Emeriaud G, Lopez-Fernandez YM, Napolitano N, Fernandez A, et al. Adherence to Lung-Protective Ventilation Principles in Pediatric Acute Respiratory Distress Syndrome: A Pediatric Acute Respiratory Distress Syndrome Incidence and Epidemiology Study. Crit Care Med. 2021;49(10):1779-89.

3. Khemani RG, Thomas NJ, Venkatachalam V, Scimeme JP, Berutti T, Schneider JB, et al. Comparison of SpO2 to PaO2 based markers of lung disease severity for children with acute lung injury. Crit Care Med. 2012;40(4):1309-16.

4. Yehya N, Harhay MO, Klein MJ, Shein SL, Pineres-Olave BE, Izquierdo L, et al. Predicting Mortality in Children With Pediatric Acute Respiratory Distress Syndrome: A Pediatric Acute Respiratory Distress Syndrome Incidence and Epidemiology Study. Crit Care Med. 2020;48(6):e514-e22.

5. Khemani RG, Smith LS, Zimmerman JJ, Erickson S, Pediatric Acute Lung Injury C. Pediatric Acute Respiratory Distress Syndrome: Definition, Incidence, and Epidemiology: Proceedings From the Pediatric Acute Lung Injury Consensus Conference. Pediatr Crit Care Med. 2015;16(5):S23-S40.

6. Ward SL, Quinn CM, Steurer MA, Liu KD, Flori HR, Matthay MA. Variability in Pediatric Ideal Body Weight Calculation: Implications for Lung-Protective Mechanical Ventilation Strategies in Pediatric Acute Respiratory Distress Syndrome. Pediatr Crit Care Med. 2018;19(12):e643-e52.

7. McLaren DS, Read WW. Classification of nutritional status in early childhood. Lancet. 1972;2(7769):146-8.

8. Fine JP, Gray RJ. A proportional hazards model for the subdistribution of a competing risk. Journal of the American statistical association. 1999;94(446):496-509.

9. Austin PC, Fine JP. Practical recommendations for reporting Fine-Gray model analyses for competing risk data. Stat Med. 2017;36(27):4391-400.

10. Yehya N, Harhay MO, Curley MAQ, Schoenfeld DA, Reeder RW. Reappraisal of Ventilator-Free Days in Critical Care Research. Am J Respir Crit Care Med. 2019;200(7):828-36.

11. Scrucca L, Santucci A, Aversa F. Regression modeling of competing risk using R: an in depth guide for clinicians. Bone Marrow Transplant. 2010;45(9):1388-95.

**Additional File 1 Table 1:** Multivariable Analysis for Secondary Outcomes of 28-day VFD (IMV and NIV) and Time to Extubation in Survivors.

|  | 28-day VFD (IMV and NIV)  (n=304) | | Time to Extubation (Survivors) (n=255) | |
| --- | --- | --- | --- | --- |
|  | SHR (95% CI) | p value | HR (95% CI) | p value |
| **Univariable Models** |  |  |  |  |
| Mechanical Power (per 0.1 J∙min^-1^∙Kg^-1^ predicted body weight) | 0.89 (0.85, 0.94) | <0.0001 | 0.96 (0.91, 1.00) | 0.067 |
| **Multivariable Models** |  |  |  |  |
| Mechanical Power (per 0.1 J∙min^-1^∙Kg^-1^ predicted body weight) | 0.92 (0.87, 0.98) | 0.006 | 0.96 (0.90, 1.02) | 0.19 |

All models are adjusted for center-level effects. Multivariable models control for the pre-specified pediatric acute respiratory distress syndrome (PARDS) severity of illness model, height, PCO_2_, and mode of ventilation. The pre-specified PARDS severity of illness model adjusts for immunocompromised conditions, the 6-hour PaO_2_/FiO_2_ ratio, and the fluid balance, vasopressor-inotrope score, and organ dysfunction on the first day of PARDS. There were two children missing length of ventilation excluded from these models.

VFD: ventilator-free days. IMV: invasive mechanical ventilation. NIV: non-invasive mechanical ventilation. SHR: subdistribution hazard ration. CI: confidence interval.

**Additional File 1 Table 2:** Propensity Score Multivariable Model for use of High Mechanical Power (≥0.62 J∙min^-1^∙Kg^-1^ predicted body weight)

|  | OR (95% CI) | p value |
| --- | --- | --- |
| Pre-Specified PARDS Severity of Illness Score Quartiles | | |
| Quartile 1  Quartile 2  Quartile 3  Quartile 4 | ref  0.05 (0.003, 0.70)  2.45 (0.43, 13.94)  5.67 (0.99, 32.39) | 0.03  0.31  0.051 |
| Median 24-hour Oxygenation Index | 1.07 (0.92, 1.27) | 0.38 |
| Pressure Controlled Mode of Ventilation | 4.48 (1.97, 10.17) | <0.0001 |
| Corticosteroids at PARDS Diagnosis | 2.04 (1.02, 4.08) | 0.044 |
| Height Quartiles | | |
| <62 cm  62-84.5  84.5-126  ≥126 | ref  0.70 (0.30, 1.60)  0.26 (0.10, 0.66)  0.10 (0.03, 0.28) | 0.40  0.005  <0.0001 |
| Comorbidity of Bronchopulmonary Dysplasia | 11.99 (2.50, 57.53) | 0.002 |
| Pre-Specified PARDS Severity of Illness Score x Oxygenation Index | | |
| Quartile 1  Quartile 2  Quartile 3  Quartile 4 | ref  1.34 (1.03, 1.75)  0.98 (0.81, 1.18)  0.96 (0.81, 1.15) | 0.029  0.82  0.69 |

n=302 children (missing data on corticosteroids n=1, median 24-hour oxygenation index n=3). The pre-specified pediatric acute respiratory distress syndrome (PARDS) severity of illness score includes variables for immunocompromised conditions, the 6-hour PaO_2_/FiO_2_ ratio, and the fluid balance, vasopressor-inotrope score, and organ dysfunction on the first day of PARDS.

**Additional File 1 Table 3:** Additional Sensitivity Analyses Limited to the Subgroup of Children <2 years of age

|  | 28-day VFD (IMV) | |
| --- | --- | --- |
|  | SHR (95% CI) | p value |
| **Multivariable Model 1:** Age <2 years old (excluding children with either bronchopulmonary dysplasia or prematurity <32 weeks estimated gestational age) (n=121) | | |
| Mechanical Power (per 0.1 J∙min^-1^∙Kg^-1^ predicted body weight) | 0.88 (0.81, 0.97) | 0.009 |
| **Model 2:** Age <2 years old (inverse probability weighted on propensity score for use of high mechanical power in children <2 years old) (n=148) | | |
| Mechanical Power (per 0.1 J∙min^-1^∙Kg^-1^ predicted body weight) | 0.92 (0.85, 0.999) | 0.047 |

All models are adjusted for center-level effects. Model 1 controls for the pre-specified pediatric acute respiratory distress syndrome (PARDS) severity of illness score, height, PCO_2_, and mode of ventilation. The pre-specified PARDS severity of illness score adjusts for immunocompromised conditions, the 6-hour PaO_2_/FiO_2_ ratio, and the fluid balance, vasopressor-inotrope score, and organ dysfunction on the first day of PARDS. Propensity score for use of high mechanical power in children <2 years old included variables for region, a comorbidity of bronchopulmonary dysplasia, PCO_2_, median 24-hour Oxygenation Index, and the PARDS severity of illness score. There were two children missing age and 1 child missing length of ventilation not included in these models. VFD: ventilator-free days. IMV: invasive mechanical ventilation.

**Additional File 1 Table 4:** Multivariable models for the association between Mechanical Energy and 28-day Ventilator-Free Days and Mortality

|  | 28-day VFD IMV (n=304) | | ICU Mortality (n=306) | |
| --- | --- | --- | --- | --- |
|  | SHR (95% CI) | p value | OR (95% CI) | p value |
| **Entire Cohort** | | | | |
| Mechanical Energy Quartiles (J∙Kg^-1^ predicted body weight) | | | | |
| <0.0132  0.0132-0.018  0.018 – 0.023  ≥0.023 | ref   - 1. (0.76, 1.34)   0.62 (0.48, 0.79)  0.65 (0.47, 0.92) | 0.94  <0.0001  0.014 | ref  0.72 (0.18, 2.84)  2.22 (0.66, 7.50)  2.08 (0.60, 7.28) | 0.64  0.20  0.25 |
| **Subgroup Age <2 years (n=149)** | | | | |
| Mechanical Energy Quartiles (J∙Kg^-1^ predicted body weight) | | | | |
| <0.0132  0.0132-0.018  0.018 – 0.023  ≥0.023 | ref  0.92 (0.53, 1.27)  0.44 (0.29, 0.67)  0.48 (0.26, 0.86) | 0.37  <0.0001  0.014 | ref  1.12 (0.10, 12.2)  7.23 (1.04, 50.3)  6.06 (0.93, 39.4) | 0.93  0.046  0.059 |
| **Subgroup Age ≥2 years (n=153)** | | | | |
| Mechanical Energy Quartiles (J∙Kg^-1^ predicted body weight) | | | | |
| <0.0132  0.0132-0.018  0.018 – 0.023  ≥0.023 | ref  1.17 (0.66, 2.08)  0.81 (0.49, 1.32)  0.86 (0.39, 1.89) | 0.59  0.40  0.70 | ref  0.26 (0.04, 1.74)  0.63 (0.10, 3.79)  0.44 (0.06, 2.96) | 0.17  0.61  0.40 |

All models control for the pre-specified pediatric acute respiratory distress syndrome (PARDS) severity of illness model, height, PCO_2_, mode of ventilation, and a comorbidity of bronchopulmonary dysplasia. The pre-specified PARDS severity of illness model adjusts for immunocompromised conditions, the 6-hour PaO_2_/FiO_2_ ratio, and the fluid balance, vasopressor-inotrope score, and organ dysfunction on the first day of PARDS. There were two children missing age not included in the age subgroup models. There were 2 children missing length of ventilation not included in the VFD models. VFD: ventilator-free days. IMV: invasive mechanical ventilation.

**Additional File 1 Table 5:** The Univariable Association between each Ventilation Management Component of Mechanical Power and 28-day Ventilator-Free Days

|  | 28-day VFD (IMV) (n=304) | |
| --- | --- | --- |
|  | SHR (95% CI) | p value |
| **Model 1: Tidal Volume Quartiles** | | |
| ≤6.25 ml/kg predicted body weight  >6.25 and ≤7.47 ml/kg predicted body weight  >7.47 and ≤9.19 ml/kg predicted body weight  >9.19 ml/kg predicted body weight | ref  0.83 (0.63, 1.09)  0.78 (0.59, 1.02)  0.74 (0.56, 0.99) | 0.18  0.068  0.042 |
| **Model 2: Delta Pressure Quartiles** | | |
| ≤15.1 cm H_2_O  >15.1 and ≤18.6 cm H_2_O  >18.6 and ≤22.1 cm H_2_O  >22.1 cm H_2_O | ref  0.87 (0.63, 1.20)  0.69 (0.47, 1.00)  0.65 (0.48, 0.88) | 0.39  0.052  0.005 |
| **Model 3: Peak Inspiratory Pressure Quartiles** | | |
| <22 cm H_2_O  ≥22 and <26 cm H_2_O  ≥26 and <31 cm H_2_O  ≥31 cm H_2_O | ref  0.89 (0.64, 1.23)  0.77 (0.54, 1.10)  0.49 (0.33, 0.73) | 0.48  0.15  <0.0001 |
| **Model 4: Respiratory Rate Quartiles** | | |
| ≤19.5 bpm  >19.5 and ≤24.5 bpm  >24.5 and ≤30 bpm  >30 bpm | ref  1.20 (0.87, 1.66)  0.96 (0.71, 1.30)  0.97 (0.69, 1.37) | 0.26  0.79  0.88 |
| **Model 5: Positive End-Expiratory Pressure Quartiles** | | |
| <6 cm H_2_O  ≥6 and <8 cm H_2_O  ≥8 and <10 cm H_2_O  ≥10 cm H_2_O | 0.82 (0.52, 1.27)  ref  0.79 (0.56, 1.12)  0.61 (0.43, 0.87) | 0.37  ref  0.18  0.006 |

All models control for center-level effects. There were 2 children missing length of ventilation not included in these models. VFD: Ventilator-Free Days. IMV: Invasive Mechanical Ventilation.

**Additional File 1 Table 6:** Multivariable Model for 28-day Ventilator-Free Days considering all Ventilator Management Components of Mechanical Power (with Delta Pressure)(n=304)

|  | 28-day VFD (IMV) | |
| --- | --- | --- |
| **Multivariable Model** | SHR (95% CI) | p value |
| Delta Pressure Quartiles  ≤15.1 cm H_2_O  >15.1 and ≤18.6 cm H_2_O  >18.6 and ≤22.1 cm H_2_O  >22.1 cm H_2_O | ref  0.99 (0.71, 1.38)  0.76 (0.53, 1.08)  0.77 (0.55, 1.09) | 0.94  0.12  0.14 |
| Tidal Volume Quartiles  ≤6.25 ml/kg predicted body weight  >6.25 and ≤7.47 ml/kg predicted body weight  >7.47 and ≤9.19 ml/kg predicted body weight  >9.19 ml/kg predicted body weight | ref  1.03 (0.81, 1.33)  0.91 (0.69, 1.21)  0.94 (0.67, 1.33) | 0.79  0.52  0.73 |
| Respiratory Rate Quartiles  ≤19.5 bpm  >19.5 and ≤24.5 bpm  >24.5 and ≤30 bpm  >30 bpm | ref  1.59 (1.03, 2.45)  1.44 (0.95, 2.17)  1.43 (0.83, 2.46) | 0.037  0.084  0.20 |
| Positive End-Expiratory Pressure Quartiles  <6 cm H_2_O  ≥6 and <8 cm H_2_O  ≥8 and <10 cm H_2_O  ≥10 cm H_2_O | 0.89 (0.60, 1.32)  ref  0.89 (0.63, 1.25)  0.78 (0.58, 1.04) | 0.55  ref  0.50  0.093 |

Multivariable model controls for center-level effects, the pre-specified pediatric acute respiratory distress syndrome (PARDS) severity of illness score, height, PCO_2_, and comorbidity of bronchopulmonary dysplasia. The pre-specified PARDS severity of illness score adjusts for immunocompromised conditions, the 6-hour PaO_2_/FiO_2_ ratio, and the fluid balance, vasopressor-inotrope score, and organ dysfunction on the first day of PARDS. There were 2 children missing length of ventilation not included in these models. VFD: Ventilator-Free Days. IMV: Invasive Mechanical Ventilation.

**Additional File 1 Table 7:** Multivariable Model for 28-day Ventilator-Free Days considering all Ventilator Management Components of Mechanical Power (with Peak Inspiratory Pressure)(n=304)

|  | 28-day VFD (IMV) | |
| --- | --- | --- |
| **Multivariable Model** | SHR (95% CI) | p value |
| Peak Inspiratory Pressure Quartiles  <22 cm H_2_O  ≥22 and <26 cm H_2_O  ≥26 and <31 cm H_2_O  ≥31 cm H_2_O | ref  0.98 (0.71, 1.36)  0.92 (0.66, 1.29)  0.67 (0.46, 0.97) | 0.91  0.62  0.033 |
| Tidal Volume Quartiles  ≤6.25 ml/kg predicted body weight  >6.25 and ≤7.47 ml/kg predicted body weight  >7.47 and ≤9.19 ml/kg predicted body weight  >9.19 ml/kg predicted body weight | ref  1.05 (0.83, 1.34)  0.92 (0.70, 1.21)  0.95 (0.66, 1.37) | 0.67  0.55  0.77 |
| Respiratory Rate Quartiles  ≤19.5 bpm  >19.5 and ≤24.5 bpm  >24.5 and ≤30 bpm  >30 bpm | ref  1.53 (0.99, 2.36)  1.43 (0.95, 2.14)  1.51 (0.86, 2.62) | 0.053  0.087  0.15 |
| Positive End-Expiratory Pressure Quartiles  <6 cm H_2_O  ≥6 and <8 cm H_2_O  ≥8 and <10 cm H_2_O  ≥10 cm H_2_O | 0.87 (0.60, 1.27)  ref  0.92 (0.65, 1.31)  0.88 (0.65, 1.20) | 0.48  ref  0.66  0.43 |

Multivariable model controls for center-level effects, the pre-specified pediatric acute respiratory distress syndrome (PARDS) severity of illness score, height, PCO_2_, and comorbidity of bronchopulmonary dysplasia. The pre-specified PARDS severity of illness score adjusts for immunocompromised conditions, the 6-hour PaO_2_/FiO_2_ ratio, and the fluid balance, vasopressor-inotrope score, and organ dysfunction on the first day of PARDS. There were 2 children missing length of ventilation not included in these models. VFD: Ventilator-Free Days. IMV: Invasive Mechanical Ventilation.

**Additional File 1 Table 8:** Structural Equation Modeling

| **Mediator** |  | **Outcomes** | | | | |
| --- | --- | --- | --- | --- | --- | --- |
|  |  |  | 28-day Ventilator-Free Days (IMV) (n=304) | | ICU Mortality (n=306) | |
|  | Variable | Effect | β Coefficient (95% CI) | p value | Odds Ratio (95% CI) | p value |
|  | Positive End-Expiratory Pressure | Indirect | -1.03 (-3.83, -0.26) | 0.106 | 0.09 (-0.001, 0.25) | 0.11 |
|  |  | Total | -12.05 (-20.76, -5.79) | <0.001 | 2.04 (1.07, 3.57) | <0.001 |
|  | Tidal Volume (per predicted body weight) | Indirect | 0.94 (-0.77, 3.64) | 0.46 | -0.08 (-0.41, 0.08) | 0.54 |
|  |  | Total | -10.98 (-17.89, -5.19) | <0.001 | 1.84 (0.78, 2.96) | <0.001 |
|  | Delta Pressure | Indirect | 0.42 (-2.07, 4.18) | 0.81 | -0.06 (-0.88, 0.45) | 0.84 |
|  |  | Total | -11.19 (-18.89, -5.29) | <0.001 | 1.93 (1.11, 3.19) | <0.001 |
|  | Respiratory Rate | Indirect | 5.27 (-4.98, 17.49) | 0.8 | -1.15 (-2.65, -0.11) | 0.08 |
|  |  | Total | -9.89 (-17.52, -3.21) | 0.006 | 1.8 (0.33, 2.77) | 0.001 |
|  | Peak Inspiratory Pressure | Indirect | -1.98 (-5.15, 2.34) | 0.28 | 0.09 (-0.45, 1.21) | 0.8 |
|  |  | Total | -11.85 (-17.98, -5.82) | <0.001 | 2.02 (0.68, 2.83) | <0.001 |

The indirect effect quantifies the mediation effect. The total effect is the sum of all indirect effects of the mediator and the direct effect of mechanical power on the outcome. 95% CI: bia-corrected and accelerated confidence intervals using 100 bootstrapped samples. There were 2 children missing length of ventilation not included in the VFD models. VFD: ventilator-free days. IMV: invasive mechanical ventilation.

**SUPPLEMENTAL FIGURE**


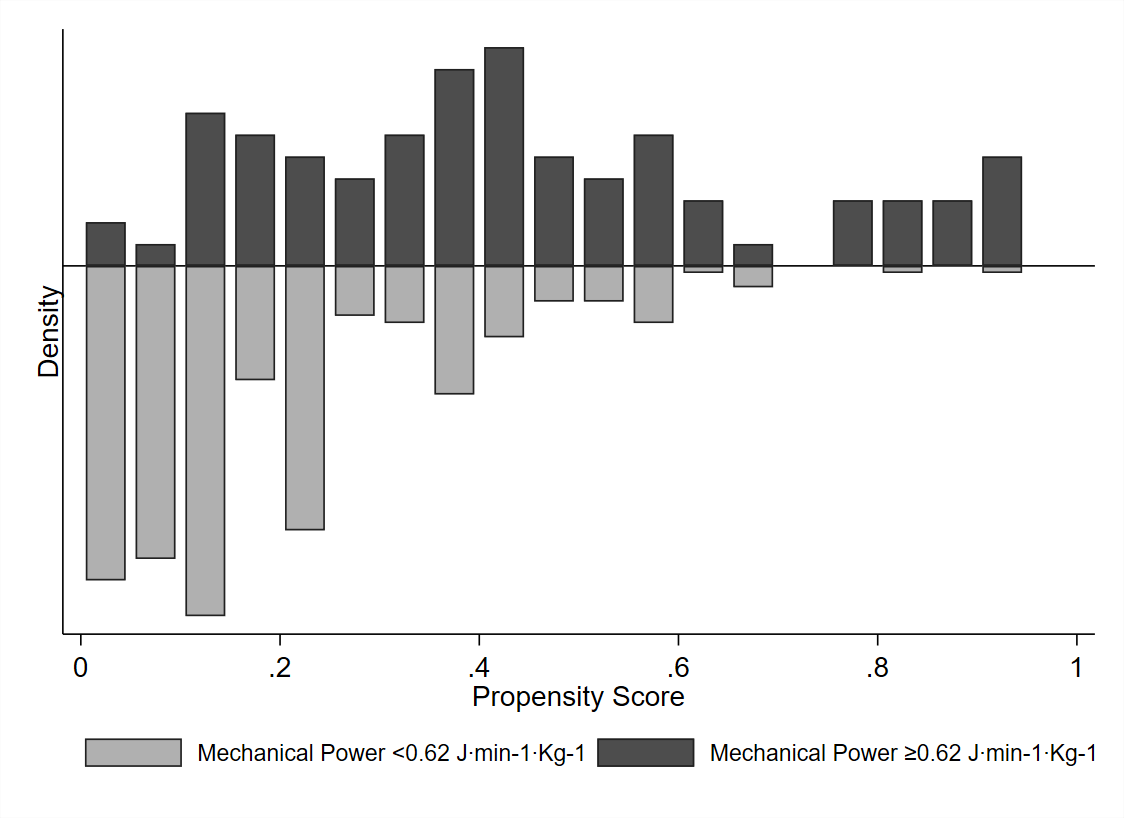


**Additional File 1 Figure 1**: Distribution of Propensity Scores
